# Tracking pollen tube and ovule development *in vivo* reveals rapid responses to pollination in *Brassica napus*

**DOI:** 10.1093/aobpla/plaf002

**Published:** 2025-01-20

**Authors:** Catherine Parry, Colin Turnbull, Richard J Gill

**Affiliations:** Georgina Mace Centre for the Living Planet, Department of Life Sciences, Imperial College London, Silwood Park campus, Buckhurst Road, Ascot, Berkshire, SL5 7PY, UK; Department of Life Sciences, Imperial College London, South Kensington campus, Exhibition Road, London, SW72AZ, UK; Department of Life Sciences, Imperial College London, South Kensington campus, Exhibition Road, London, SW72AZ, UK; Georgina Mace Centre for the Living Planet, Department of Life Sciences, Imperial College London, Silwood Park campus, Buckhurst Road, Ascot, Berkshire, SL5 7PY, UK

**Keywords:** *Brassica napus*, ovule, pollen tube, fertilization, pollination, controlled pollination, aniline blue

## Abstract

Pollination and subsequent fertilization in most angiosperms are precursors of seed and fruit development. Thus, understanding the developmental processes can improve the management of plant reproductive success and food security. Indeed, the window between ovule fertilization and seed development is crucial for the accumulation of metabolites which determines ultimate seed quality and yield. Establishing detailed temporal maps of development to describe pollination to early seed development is therefore extremely valuable to provide context for molecular studies, plant breeding, and to refine crop management strategies for optimal seed quality. Here, we characterize aspects of post-pollination responses in the globally important crop plant *Brassica napus* (oilseed rape, canola) with a high-resolution time series of microscope images of the floral organs during the first 48 h post pollination. We demonstrate the rapid response to pollination in *B. napus* (c.v. Westar), with pollen tubes germinating and traversing the style within just 4 h. We also describe markers of early seed formation in response to fertilization in the synchronous development of ovule area and stigma length. Our results provide a series of temporal benchmarks for post-pollination floral morphology in *B. napus*, representing valuable reference points for studying and tracking pollination responses and early seed development.

## Teaser

Establishing detailed temporal maps to describe development is valuable to provide context for studies of pollination and seed development. Here, we present a high-resolution time series of microscope images of the floral organs of *Brassica napus* (oilseed rape) post pollination. Our characterization of pollination responses demonstrates the rapid response to pollination in *B. napus* , and provides a reference for future studies.

## Introduction

Pollination represents a vital milestone in plant development, underpinning the genetic health of plant communities (Rodger *et al.* 2021), and having significant ramifications for food security (Woodcock *et al.* 2019; Ollerton 2021). Given its importance for sexual reproduction in angiosperms, efforts to characterize the developmental process that enables pollination and fertilization remains an active research area (Song *et al.* 2021). The progamic phase—the time between pollination and ovule fertilization—is a complex developmental period during which compatibility is determined, pollen tubes are established, and substantial hormonal and chemical signalling occurs between pollen and pistil to ensure successful fertilization (Dresselhaus *et al.* 2016). This process is fundamental to plant fitness, therefore understanding the developmental dynamics of pollination and early seed development has important applications for ecology and agronomy (Fairhurst *et al.* 2022).

A defining feature of the progamic phase is pollen tube growth, which is a key determinant of an individual plant’s reproductive success (Zakharova *et al.* 2022). The pollen tube is a distinctive cell structure that comprises one of the most rapidly growing cell types in plants (Adhikari *et al.* 2020a). Yet, growth rate is variable across taxa, ranging from 10 µm per hour to over 20 mm per hour at their extremes (Williams 2012; Williams *et al.* 2016). Pollen tube growth rates are thought to be related to genome size and ploidy level, given the physical burden of sperm cells, but there is some degree of energetic compensation in terms of the rates of callose deposition, cell wall construction, and thickness, and the overall metabolic rate of the vegetative tissues (Williams 2012; Williams and Oliveira 2020). In experimental settings, rates of pollen tube growth are influenced by the matrix in which they grow (*in vivo*, or the choice of *in vitro* growth medium) (Roeder *et al.* 2021), and environmental conditions, especially temperature and humidity (Rozier *et al.* 2020). Moreover, complex inter- and intra-specific interactions occur among gametophytes which may be in direct competition in the style (Williams and Reese 2019). Thus, general predictions of pollen tube growth across plant species are likely to be error-prone unless directly quantified.

Seed production and high yield in seed crops are contingent on the successful fertilization of the ovule, ensured by the pollen tube, which ultimately functions to deliver the male gametes contained in the sperm cell(s) to the female gametes (egg) (Bleckmann *et al.* 2014). Once the pollen tube arrives at the ovule, guided by calcium ion gradients and chemical attractants, it penetrates the micropyle, and bursts to release the two sperm cells for double fertilization (Adhikari *et al.* 2020b). Fertilization marks the initiation of seed and fruit development, triggering a succession of developmental changes which gives rise to the endosperm and embryo (Dresselhaus and Franklin-Tong 2013). This delineates a shift in development, with significant upregulation of auxin and gibberellic acid metabolism to promote the cell division and expansion necessary for development of the seed and fruit (Dorcey *et al.* 2009). This period of cell differentiation is energetically demanding; initially the embryo is provided with nutrients and growth factors by the transient endosperm, and then the maternal tissue (Bleckmann *et al.* 2014). These accumulations will eventually become seed reserves, and determine the ultimate size of the seed, and thus represent a crucial time for seed development (Song *et al.* 2021).

Documented rates of species-specific pollination responses and post-pollination development provide a highly relevant resource to contextualize studies of pollination, fertilization, and development of seed and fruit. Quantifying this for important plants, such as world-leading food crops, has wide-ranging applications in agriculture and horticulture (Dadlani and Yadava 2023). Establishing the rate at which developmental responses to pollination occur provides a temporal reference for the study of molecular mechanisms, and for the exploration of potential physiological indicators of plant development and pollination status (Parry *et al.* 2024).

Here we provide a characterization of pollen tube growth and floral organ morphology, *in vivo*, and at high temporal resolution. We monitored development shortly after hand-pollination until petal drop in the pollinator dependent, economically and calorically important crop plant *Brassica napus* (a.k.a. canola, oilseed rape). We demonstrate that the development of pollen tubes from germination to fertilization is rapid, within just 4 h. On fertilization, fertilized ovule area and stigma length increase rapidly and in concert post pollination, marking preparations for early seed development and nutrient assimilation.

## Methods

### Plant pollination trial and tissue sampling

*Brassica napus* plants of the spring variety, Westar, were grown from seed to flowering stage in a controlled environment (CE) room under ‘long day’ conditions (16 h day at 22°C with 180 µmol m^-2^ s^-1^ light intensity (under 4000K Cool White Fluorescent lamps), 8 h night at 18°C, both under 60% relative humidity). Seeds were sown in Levington F2S Compost medium and cold-stratified for 4 days at 4°C before being transferred to the CE room. Seedlings were transplanted into 9 cm pots containing Levington M3 Compost medium and grown to six-true leaf stage at which they were transplanted finally into 11-litre capacity deep pots. Plants were kept well-watered throughout, and from the six-true leaf stage 500 ml fertilizer per plant (prepared by adding 1.7 ml of Miracle-Gro fertilizer to 500 ml water) was added to watering every 2 weeks.

The pollination trial began when the first flower opened, approximately 8 weeks after sowing. On opening, each flower was emasculated (stamens removed before anthesis with forceps) and labelled with an ID collar (a segment cut from a plastic straw) around the peduncle. Within 2 h of opening, once the stigma was receptive (determined when the stigma surface became swollen with papillae), each flower was hand pollinated by brushing ample pollen directly from an anther removed from a parent plant kept in isolation (separate CE room). At specified time points post-pollination (4, 8, 12, 16, 20, 24, 28, 32, 36, 40, 44, or 48 h, respectively) assigned flowers were removed from the plant. The style was immediately dissected and immersed in 96% ethanol until staining. We ensured that flowers were collected evenly between individual plants to prevent any time point biases across plant IDs.

### Pollen tube staining and microscopy

A stock solution of decolourized Aniline Blue (DAB) was prepared by dissolving aniline blue in 67 mM K_2_HPO_4_ solution to 10 mg/ml. On the day of use, the solution was diluted in 67 mM K_2_HPO_4_ to achieve a final concentration of 0.1% aniline blue. This working solution was vortexed, filtered through activated charcoal to decolourize, then pure glycerol was added at 1% (v/v). The day before the plant-style tissue was to be visualized under the microscope, each sample was prepared by rehydrating over 1 h in a serial dilution of 96% ethanol to distilled water (from 70% ethanol solution to 30% ethanol). The samples were rinsed in distilled water, and then immersed in 8 M NaOH solution for 6 h. The samples were again rinsed with distilled water and immersed in the working 0.1% DAB solution for 12 h.

To prepare the dissected styles for microscopy, each was placed on a separate microscope slide with a drop of pure glycerol then lightly squashed under a glass cover slip. Slides were protected from direct light before being visualized under a Zeiss Axiophot microscope, under fluorescence illumination with a 475–525 nm filter, and under brightfield illumination, under 50× and 100× magnification. Images were visualized and recorded with the Axiovision software and scale bars were set using a stage micrometer. Images of individual pollinated styles were taken under the microscope. Any samples damaged during preparation were excluded. Samples where aniline blue fluorescence was not sufficiently bright to accurately track pollen tubes were checked for pollen grain germination to confirm our pollination treatment but excluded from analyses. The images of the highest clarity were selected for further analysis, and a total of 89 viable images were selected, which constituted between 4 and 24 samples per time point.

### Measuring growth rates

All image processing was done with ImageJ version 2.14.0/1.54f, with images joined using the pairwise stitching tool. Contrast and brightness of the images were adjusted as necessary to improve clarity. The length of pollen tubes was measured using the line tool to measure the distance from one callose plug to the next, between the surface of the stigma and the region of first two ovules closest to the stigma. One pollen tube was measured in each style. Pollen tubes were identified by noting brightly fluorescing callose plugs regularly occurring along the style, distinctive from sieve tubes which provide some background fluorescence (Chu Chou and Harberd 1970). The ovule area was also measured for each ovule visible in the image at 100× magnification, using the elliptical tool to trace around the outer edge of each ovule.

Analysis and production of graphs was done using R Studio (version 2023.12.0 + 369), (S1-S3) with additional packages ‘ggplot2’ (Wickham 2016), ‘performance’ (Lüdecke *et al.* 2021a), and ‘see’ (Lüdecke *et al.* 2021b). For model fitting (time as a predictor of stigma length and ovule area), the performance of multiple generalized linear models (GLM) and locally estimated scatterplot moving regression (LOESS) were compared. Gaussian distribution was applied in both model types, and three link functions were tested for the GLM (normal, logarithmic, and inverse). Visual checks for normality, linearity, and homogeneity were done to ensure model assumptions were not violated, and the best fitting model was assessed by Akaike Information Criterion.

## Results

With pollination essential for *B. napus* plants to reach their yield potential, and flowering a key developmental period for management, we set out to resolve the timing of post-pollination developmental responses. To provide a temporal characterization of pollination responses we produced a high-resolution time series of microscope images of pollinated pistils stained with aniline blue. Below we report the rate at which pollen tubes germinate and traverse the style and observe the markers of early seed formation by describing the synchronous development of ovule area and stigma length.

**Figure 1.**
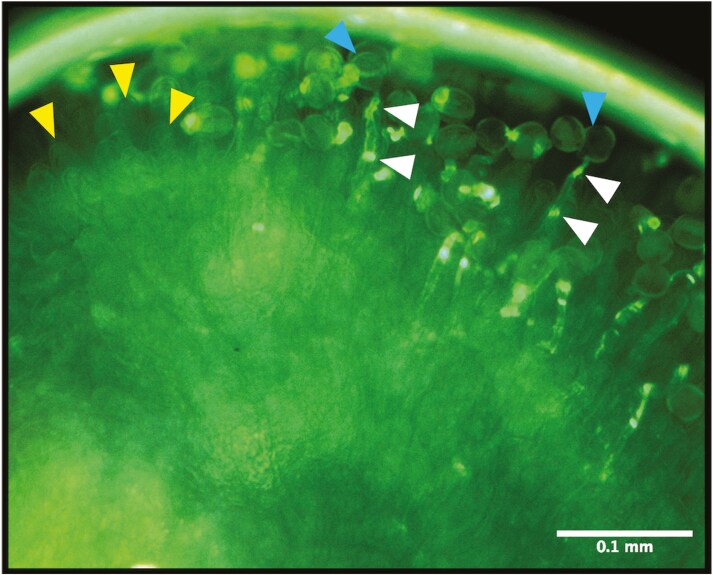
Germinating pollen grains on the stigma surface at 4 h after controlled pollination of a *Brassica napus* flower, in which they are adhered to the stigma papillae. Selected examples of papillae are pointed out using yellow arrows, a germinated pollen grain using blue arrows, and callose deposits marking the pollen tube using white arrows. Tissues were stained with aniline blue, and the image was taken using under fluorescence illumination at a magnification of 100×.

All sampled flowers had been successfully pollinated by our controlled hand-pollination protocol involving a single pollination event, as evidenced by germinated pollen grains visible at the stigma surface (Fig. 1). Pollen grains can be seen in close contact with the stigma papillae, with pollen tubes emerging from the germinated pollen grain and extending towards the transmitting tract. This confirms the effectiveness of our controlled pollination protocol, indicating both high receptivity of the style within the two first hours of flower opening and a high degree of intra-specific compatibility in this variety.

**Figure 2.**
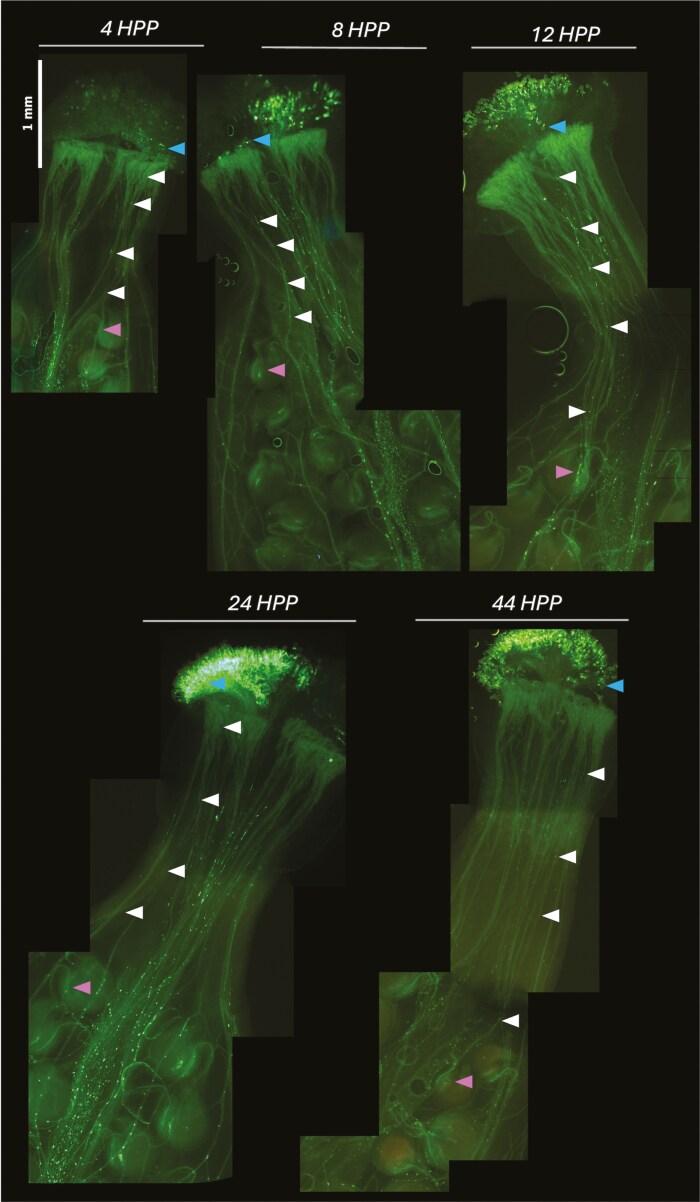
Pollen tube growth in *Brassica napus* styles. Blue arrows point out an example of a germinated pollen grain, the white arrows point to the growth of an example pollen tube marked by callose plugs. The pink arrows mark the pollen tubes’ point of entry at the ovule. Images show representative examples of styles imaged at 4, 8, 12, 24, and 44 h post-pollination. Tissues were stained with aniline blue and the image was taken using under fluorescence illumination at a magnification of 100×. Multiple images were joined to comprise each style image using the ImageJ pairwise stitching tool.

**Figure 3.**
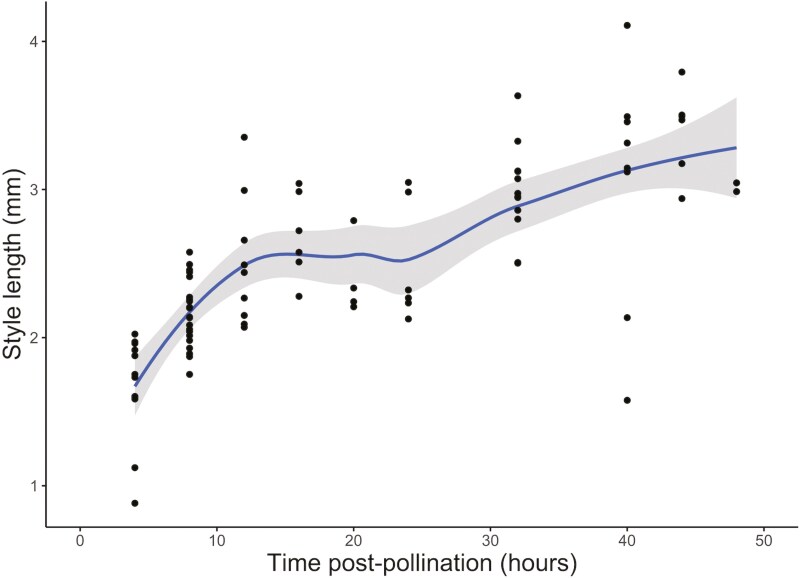
Growth of styles measured from stigma tip to first two ovules in *Brassica napus* flowers after a controlled pollination event. The grey ribbon denotes the 95% confidence interval of the LOESS model. Each data point is a measured style from a high-quality image of an individual flower.

**Figure 4.**
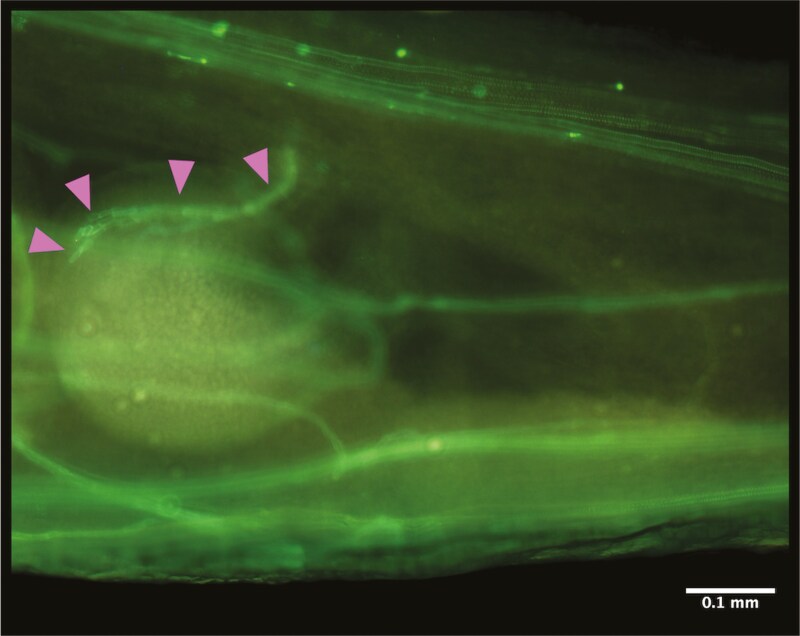
Ovule in an ovary of a *Brassica napus* flower 4 h post pollination. Pink arrows point to callose deposition at pollen tube entry into the ovule. Tissues were stained with aniline blue and the image was taken using under fluorescence illumination at a magnification of 100×.

Following pollen grain germination, we found pollen tubes to develop rapidly, growing at a high rate through the transmitting tract to reach the ovule region within 4 h post-pollination (Fig. 2). Thus, all further measures of style length can be considered post fertilization. Mean (± standard error) style length measured 1.67 ± 0.35 mm at our first-time point of 4 h post pollination, equating to a mean estimated pollen tube growth rate of at least 400 μm per hour (Fig. 3). Our images show pollen tubes grew in a highly directional manner through the transmitting tract down the length of the style (Fig. 2). Once a pollen tube reached the region of an ovule, the tube showed a distinct change in direction towards the micropyle, before entering the ovule, where further callose is deposited as the micropyle is penetrated for fertilization (Fig. 4).

**Figure 5.**
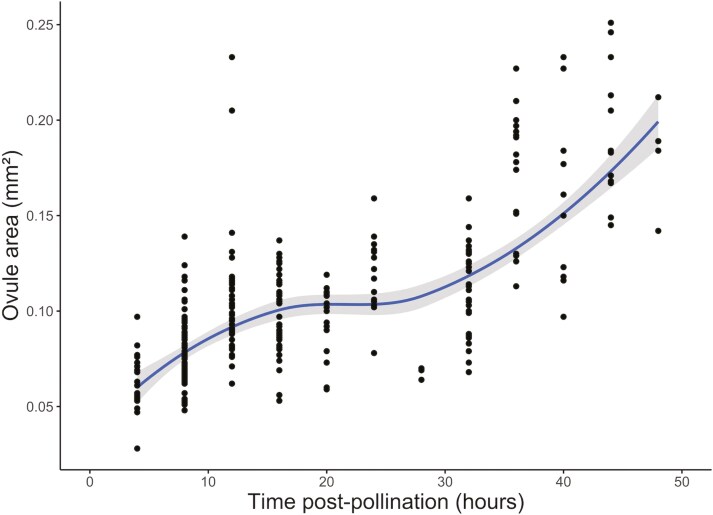
Growth of ovule area in *Brassica napus* flowers after a controlled pollination event. Ovules were measured as 2D surface area. The grey ribbon denotes the 95% confidence interval of the LOESS model.

Interestingly, style length continues to increase post fertilization in an approximately sigmoidal pattern, where the rate of lengthening is high between 4 and 12 h, and slows until between 24 and 48 h post pollination, after which the rate of lengthening accelerates again (Fig. 3). This non-linear rate of growth is mirrored in development of the ovules, whose area expands with time post pollination. Ovule area expands rapidly between 4 and 18 h post pollination, with the rate of expansion slowing until 32 h post-pollination after which the average ovule area rapidly expanded (Fig. 5). Ovule expansion was consistently observed across all samples imaged with no underdeveloped ovules, again reiterating the effectiveness of our controlled pollination treatment.

## Discussion

Here, we characterized and quantified the rate at which the gynoecia of *B. napus* flowers develop in response to controlled pollination *in vivo* at a high temporal resolution. From pollen deposition to petal drop, we found that germination of pollen grains occurs rapidly on the stigma, and that pollen tubes grow to reach the ovules within just 4 h of pollen deposition. Following micropyle penetration by a pollen tube, ovules rapidly begin to swell, with their two-dimensional area increasing in two phases between 4 and 16 h after pollination, and again from 32 h. Interestingly, the length of the stigma increases in parallel to ovule expansion, both in a general sigmoidal pattern. Our findings highlight the important window in which the key pollination and fertilization processes are initiated in this crop plant, and offer a temporal period to which pollination biomarkers can be explored.

The rapid physiological response to pollination in *B. napus* is initiated by the adherence of pollen grains to the stigma papillae, where they hydrate, germinate, and develop pollen tubes in quick succession (Fig. 1). We tracked each pollen tube’s development by marking its growth from one callose deposition to the next until it reached the ovule closest to the style (this is shown to be the first ovule fertilized (Jie and Jiaheng 2007)). Our results confirm previous reports of rapid adhesion, hydration, and germination of a compatible *B. napus* pollen grain within 30 min of deposition (Zhang *et al.* 2017). While reports suggest the rate of growth in *B. napus* pollen tubes is greater than other *Brassicaceae* genotypes (Hill and Lord 1986), and there is significant variation between cultivars and pollen parent genotype (Ockendon and Gates 1975), our estimated 400 μm per hour is in line with previous reports (Ockendon and Gates 1975; Hill and Lord 1986; Jie and Jiaheng 2007). Pollen tubes can be seen to extend in a highly directional manner throughout the transmitting tract (Fig. 2). The rapid establishment and growth of the pollen tubes are essential for the delivery of the male gametes contained in the sperm cell to the female gametes (egg cell). The development of the pollen tube is supported by the transmitting tract tissue which contributes to the guidance of the pollen tube through provision of nutrients and signalling molecule gradients along its length (Pereira *et al.* 2021). Once the pollen tube reaches the vicinity of the ovule, the synergid cells of the ovule become dominant attractants, releasing diffusible signals including small cysteine-rich proteins which guide the pollen tube precisely to the micropyle (Kessler and Grossniklaus 2011). Consistent with this observation, pollen tubes can be seen to deviate from their linear growth pattern, changing orientation towards micropylar aperture in the present study (Fig. 2).

On reaching the ovule, the pollen tubes penetrate the micropyle, continuing to deposit callose, and growing towards the synergid cells of the ovule for fertilization (Fig. 4). From 4 h post pollination, ovule area expands in two phases, first, between 4 and 18 h, and second, from 32 h (Fig. 5). In *Arabidopsis thaliana,* it has been shown that receipt of pollen tube contents (analogous to seminal plasma) induces ovule enlargement via cell division and cell expansion within the first 24 h post-pollination (Kasahara *et al.* 2016). This process, known as pollen tube-dependent ovule enlargement morphology is thought to be a precursor to the initiation of development of the seed coat, before double fertilization (Kasahara *et al.* 2017). Although we cannot determine the precise moment of double fertilization from our results, other reports of *B. napus* suggest this occurs within 18–24 h (Pechan 1988; Jie and Jiaheng 2007). During the second phase of ovule expansion, it is therefore likely that double fertilization is complete, and thus both cell expansion and division are occurring at a high rate towards the early embryo development and expansion of the primary endosperm (Jie and Jiaheng 2007). Concurrently with ovule expansion, from 4 h post pollination the length of the style increases (Fig. 3). Style elongation post-pollination could be an artefact of the completion of floral development, distinct to the growth of ovule area, but we postulate it to be a trait of replum formation as an early element of seed pod development (van der Knaap and Østergaard 2018).

Ovule expansion and style lengthening represent early seed development, a critical period for seed viability and ultimately crop yields, wherein the endosperm expands and the integument is defined as the early seed coat (Rolletschek *et al.* 2021). Here, the ovule becomes a significant sink for resources, as sucrose and starch are accumulated by the endosperm, and embryo begins to develop (Li and Berger 2012; Dadlani and Yadava 2023). The accumulation of metabolites are crucial determinant of seed size, protein, and oil content (Li and Berger 2012), while seed development can be vulnerable to stress, environmental perturbation, and availability of nutrients during this time (Kumar *et al.* 2021). The ovule expansion seen here (Fig. 5) suggests resource assimilation begins immediately from the point of fertilisation, so the post-pollination window must be considered as a particularly important period for the management of agricultural inputs to optimize seed quality and yield.

## Conclusion

Our characterization of post-pollination responses in the globally important crop *B. napus* provides a temporal reference for morphological indicators during the first 48 h of embryo development. Understanding post-pollination development has wide-ranging applications in agriculture and horticulture, particularly for pollinator-dependent crops, whose yield and quality are reliant on the delivery of adequate pollen (Bailes *et al.* 2015). Given the importance of high pollination rates for *B. napus* and many other crops to reach yield potential, understanding post-pollination floral organ morphology will be important for the ongoing development of pollination monitoring methods.

## Supplementary Material

plaf002_suppl_Supplementary_Datasets_1-3

## Data Availability

The data underlying this article are available in the article and in its online supplementary material. Original images from which this data were derived will be shared by the corresponding author on reasonable request.

## Notes

CP was supported by an Imperial College London departmental PhD studentship, and by funding from a BBSRC PhenomUK research grant (BB/Y512333/1). RJG was supported by the Quekett Microscopical Club.
