# Supplementary material for: Tracking pollen tube and ovule development in vivo reveals rapid responses to pollination in Brassica napus
Source: AoB Plants. 2025 Jan 20;17(2):plaf002. doi: 10.1093/aobpla/plaf002 (PMC11880539; doi:10.1093/aobpla/plaf002)
Supplement: plaf002_suppl_Supplementary_Datasets_1-3 [file plaf002_suppl_supplementary_datasets_1-3.zip › aobplants-24181-TR1-s01.docx]

Department of Life Sciences

Imperial College London

Silwood Park Campus

Buckhurst Road, Ascot

Berkshire, SL5 7PY, UK

17 December 2024

Dear Dr Buckley,

Thank you for considering our research article (24181-T). We appreciate the time invested by you and the reviewers, and are pleased that you recognise the rigour of our experimental work and the contribution of our findings. Pleased find the reviewer comments and requests below (black italics) and our responses (in blue).

Kind Regards,

Catherine Parry and Richard Gill

*Reviewer 1*

*l.86 ´....potential physiological indicators of plant development and pollination status (Parry et al., 87 2024, in press). Please add the citation to the reference list. Is it perhaps this one:* [*https://doi.org/10.1111/1365-2664.14807*](https://doi.org/10.1111/1365-2664.14807)

Correct; the article has now been published and so has now been added to the bibliography.

*The Results section could have a bit more description. Now it is very short. Start the Results section with a few lines of introduction, which species used, why, what was the question/or novelty investigated.*

We have added some text to the start of the results section to provide context, setting out the species used, and the importance of the question being investigated.

It now reads: “With pollination essential for B. napus plants to reach their yield potential and flowering being a key developmental period for management, we set out to resolve the timing of post-pollination developmental responses. To provide a temporal characterisation of pollination responses we produced a high-resolution time series of microscope images of pollinated pistils stained with aniline blue. Below we report the rate that pollen tubes germinate and traverse the style and describe the markers of early seed formation by describing the synchronous development of ovule area and stigma length.”

*In the M&M section there is a mention about the number of samples analyzed, however this should be more clearly (detailed) stated (e.g., from 4 to 24 samples is a big difference).*

We have added some additional detail to explain that only samples which met our requirements for image quality were analysed. Due to the fragility of biological samples, more samples were collected than the number ultimately imaged and analysed.

It now reads: “Images of individual pollinated styles were taken under the microscope. Any samples damaged during preparation were excluded. Samples where aniline blue fluorescence was not sufficiently bright to accurately track pollen tubes were checked for pollen grain germination to confirm our pollination treatment but excluded from analyses. The images of highest clarity were selected for further analysis, with a total of 89 viable images which constituted between 4 and 24 samples per time point.”

*Please add to the legends as well this information on sample number.*

We have added additional detail.

*Reviewer 2*

*Lines 73-75: Rephrase the sentence: This period of cell differentiation is energetically expensive, during which the embryo is initially fed with nutrients and growth factors by the transient endosperm, and then the maternal tissue (Bleckmann et al., 2014)*

We have rephrased and the sentence to now read: “This period of cell differentiation is energetically demanding; initially the embryo is provided with nutrients and growth factors by the transient endosperm, and then the maternal tissue (Bleckmann et al., 2014).”

*Line 142: why such a big variation in the number of replicates? Please explain.*

Please see our response above in which the new text details why.

*Line 175: the term "self-compatibility" better applies to autogamous crosses, which is not the case in this study if I correctly understand the study design.*

We agree, and have replaced with “intraspecific compatibility” to be more accurate.
